# An extension of the technology acceptance model for understanding travelers’ adoption of variable message signs

**DOI:** 10.1371/journal.pone.0216007

**Published:** 2019-04-25

**Authors:** El Bachir Diop, Shengchuan Zhao, Tran Van Duy

**Affiliations:** School of Transportation and Logistics, Dalian University of Technology, Dalian, P.R. China; Univerza v Mariboru, SLOVENIA

## Abstract

Understanding travelers’ acceptance of Advanced Traveler Information Systems (ATIS) is crucial to the implementation of Intelligent Transportation Systems (ITS) capable of mitigating traffic congestion and improving network performance. This paper adopted an extended Technology Acceptance Model (TAM) to predict and explain road users’ intention to use Variable Message Sign (VMS) information. In addition to the traditional parsimonious TAM constructs (perceived usefulness, perceived ease of use and behavioral intention), the model examined the effects of attitude towards route diversion, familiarity with road network and information quality on road users’ acceptance of VMS. 762 drivers were interviewed and the obtained data were processed using Structural Equation Modeling. The results showed that travelers’ attitude towards route diversion had a positive effect on perceived usefulness and intention to use VMS. Information quality had a positive direct effect on perceived usefulness, perceived ease of use and attitude towards route diversion. Familiarity with the network had a positive effect on attitude towards route diversion and a negative effect on the perceived usefulness of VMS information. Perceived ease of use significantly and positively affected perceived usefulness and intention to use VMS. Perceived usefulness also had a positive effect on intention. Several academic and practical implications were also discussed.

## Introduction

As a major component of Advanced Traveler Information Systems (ATIS), Variable Message Signs (VMS) have been widely adopted to assist travelers in making more informed decisions. VMS can provide real-time information relating to speed limit, congestion, safety warnings (e.g. fogs, slippery roads, etc.), parking, etc. In China and internationally, recent technological developments have helped implement more advanced information display strategies [1–5]. In China, major cities such as Shanghai have considered the possibility of adopting more advanced VMS such as D-VMS. Unlike the type of VMS used in many Chinese cities, D-VMS can display information on both the local streets and the expressway [4,5]. However, despite the technological advancements, there is still room for improvement and the existing VMS still have not reached the effectiveness expected mainly due to travelers’ lack of acceptance of the information provided [6,7]. Thus, there is a need to investigate the factors affecting drivers’ acceptance of VMS. Identifying these factors and their effects will help better predict and explain drivers’ response to the information displayed, which, in turn, will help improve the design and implementation of VMS.

In previous studies, one of the main factors affecting drivers’ response to VMS was reliability [6,8,9]. Reliability was usually associated with degree of trust in VMS which was also found to significantly affect travelers’ compliance with VMS [7,10,11]. Clearness and understandability also played a major role in drivers’ response to VMS. In this regard, factors such as display format were important in the decision-making process. Two types of display formats were compared in the literature: graphical and text-based formats. It was found that drivers were more likely to comply with the graphical display which is easier to understand and memorize [12,13]. Relevance was also found to be a significant factor affecting drivers’ compliance with VMS. Traffic information is relevant if it has direct consequences on driver behavior. Chatterjee and McDonald [14] showed that information that reports no changes on driver’s habitual route is likely to be ignored. Network conditions such as occurrence of congestion, travel time and delay also play a major role in travelers’ response to VMS [5,15,16]. Finally, several studies also examined the effects of individual characteristics such as age, gender, familiarity with the network, income, etc. and found significant effects on drivers’ acceptance of VMS [4–7,10].

Recently, several research efforts adopted models of user acceptance of information systems to predict and explain drivers’ response to ATIS. The most commonly used framework is the Technology Acceptance Model (TAM). TAM is a simple and robust framework and has been widely adopted to explain user acceptance of information technology. TAM shows how the individual’s salient beliefs (perceived usefulness and perceived ease of use) predict his/her behavioral intention to use a given system, which, in turn, predicts his/her actual system use [17,18]. TAM has been widely adopted in different areas of research such as marketing [19,20], tourism [21], e-government [22], data sharing and virtual communities [23], etc. Recently, TAM has been adopted in the transportation research area to explain drivers’ adoption of road guidance systems. In the transportation safety sub-domain, TAM was used to explain drivers’ acceptance of distraction mitigation systems [24], on-board monitoring systems [25], and warning systems for railway level crossing [26]. In addition to the traditional TAM, their models included constructs such as unobtrusiveness, trust, perceived behavioral control and social norms. In terms of route and departure time choice, Lin et al. [27] extended the TAM to investigate road users’ adoption of web-based ATIS. They augmented TAM with information quality, response time and system accessibility. Xu et al. [28] also examined drivers’ adoption of ATIS by incorporating into their model trust, information attributes, socio-demographic characteristics and cognition of alternate routes. Other studies have also customized TAM to investigate the adoption of in-vehicle driving assistance and multimedia devices [29,30], travelers’ acceptance of autonomous cars [31], etc. They found that in addition to the TAM constructs, drivers’ adoption was also affected by variables such as perceived enjoyment, personal innovativeness, perceived behavioral control and trust.

However, among the reported studies, few have investigated travelers’ acceptance of VMS. The factors affecting travelers’ acceptance may vary depending on the road guidance system. For example, VMS provide en-route, real-time traffic information. This means that the VMS may suggest the traveler to divert from his/her current, habitual route. Thus, incorporating travelers’ attitude towards route diversion could help obtain a more accurate model. Travelers could also be advised to divert and navigate through a less familiar route. Thus, travelers’ familiarity with the road network could also affect their response to VMS. Furthermore, the quality of the information provided could also play a major in the decision-making process [27]. Finally, despite China’s fast-growing economy and car-ownership, a few studies applying TAM to Chinese drivers’ acceptance of road guidance systems have been reported internationally, to the best of the authors’ knowledge. The current study addresses the aforementioned issues by proposing an extended TAM to examine road users’ acceptance of VMS information in Dalian City, China. In addition to the traditional parsimonious TAM, the model incorporates domain-specific constructs which are information quality, attitude towards route diversion and familiarity with the road network to better understand drivers’ intention to use VMS. 762 drivers from Dalian, China were interviewed and the model was tested using Structural Equation Modeling.

The remainder of this paper is organized as follows. Section 2 describes the theoretical background and model framework used in this study. Section 3 outlines the data collection and modeling methodology. Section 4 analyzes the validity of the proposed model and tests the hypotheses formulated in Section 2. Section 5 discusses the results and their implications and concludes with study limitations and directions for future research.

## Theoretical background and research framework

### 2.1 The technology acceptance model

**Fig 1:**
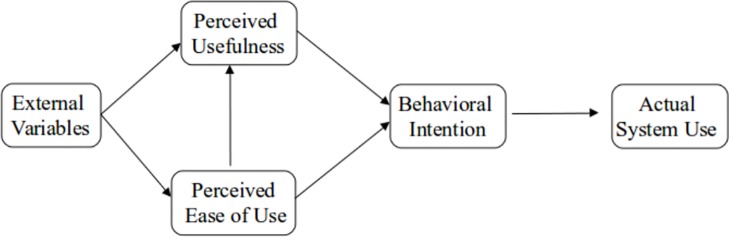
Parsimonious TAM [33]. Caption credit: Davis FD, Venkatesh V. A critical assessment of potential measurement biases in the technology acceptance model: Three experiments. International Journal of Human-Computer Studies. 1996;45(1):19–45. https://doi.org/10.1006/ijhc.1996.0040.

TAM [18] was adopted from the Theory of Reasoned Action (TRA) [32] and has been widely used to predict and explain user adoption of new information technology. TAM posits that an individual’s salient beliefs about a system (perceived usefulness and perceived ease of use) determine his/her attitude towards using the given system. Attitude is the individual’s positive or negative feelings towards a behavior [32]. Perceived usefulness is the individual’s salient belief that using a particular technology will improve his/her performance; perceived ease of use is the individual’s salient belief that using a particular technology is free of effort [18]. Furthermore, Davis [18] hypothesizes that attitude determines the individual intention to adopt the system, which in turn, determines actual system use. Intention is the individual’s perceived probability that he/she will use the system. However, Davis et al. [17] found that attitude only partially mediated the effect of perceived usefulness and perceived ease of use on behavioral intention; therefore attitude was excluded from the parsimonious TAM (Fig 1) [24,33,34].

This study uses the parsimonious TAM as foundation for our model framework. The next sub-sections describe the causal relationships among the constructs used in this study.

### 2.2 Perceived usefulness

Drawing from Davis [18], we can define perceived usefulness as the extent to which drivers believe that using VMS will improve their travel planning. In previous studies on the effects of traffic information, relevance was one of the most common indicators of the usefulness of traffic information [14]. They showed that traffic information is relevant if it has direct consequences on the road user’s behavior. Furthermore, the information should help travelers in avoiding congestion, improving their route and departure time choice and arriving on time [27]. Such information is considered useful and is likely to be accepted. Thus, we expect perceived usefulness to have a positive impact on the intention to use traffic information. This relationship was also confirmed by studies on user acceptance of information technology and road guidance systems [17,18,26,35]. Therefore, we hypothesize:

H1: Perceived usefulness has a positive effect on the intention to use VMS.

### 2.3 Perceived ease of use

In the context of drivers’ response to traffic information, perceived ease of use refers to concepts such as legibility, understandability and ease of learning [18]. Studies on information display format showed that legibility and understandability are major factors affecting drivers’ acceptance of ATIS [12,13,36]. Thus, we can assume a strong relationship between perceived ease of use and intention to use VMS. Results from previous TAM studies also suggest a positive relationship between perceived ease of use and intention [17,28,30].

Furthermore, Perceived ease of use has a positive effect on perceived usefulness. An ATIS that is easy to use will require less processing time and effort. Thus, the driver can use the information in less time and turn his/her focus to other driving tasks, which can improve his/her driving performance. This argument is also corroborated by previous studies on drivers’ acceptance of road guidance systems [24,25,29]. Therefore, we propose:

H2: Perceived ease of use has a positive effect on the intention to use VMS.
H3: Perceived ease of use has a positive effect on the perceived usefulness of VMS.

### 2.4 Attitude towards route diversion

Earlier TAM studies found that *attitude towards using the system* only partially mediated the effect of perceived usefulness and perceived ease of use on intention and removed it from the parsimonious TAM [24,33,34]. Thus, this study adopts a concept of attitude that is more specific to travelers’ route switching behavior in response to VMS: *attitude towards route diversion*. Studies in behavioral science defined attitude as the individual’s positive or negative feelings towards a given behavior [32]. Therefore, we define attitude towards route diversion as the road user’s positive or negative feelings towards route diversion.

Studies on route choice behavior have shown that certain drivers exhibit habitual behavior and choose the same route regardless of the current conditions [37]. Bogers et al. [37] argue that when travelers exhibit habit, the provision of traffic information is less likely to change their behavior. In other words, they do not find the information useful. Unlike habitual behavior, route diversion is likely to involve the acquisition and assessment of traffic information on the available routes. For this reason, travelers with positive attitude towards route diversion are more likely to seek the information that can help them evaluate the current traffic conditions and choose their route accordingly. Thus, it is rational to assume that travelers with positive attitude towards route diversion would find VMS information more useful and would be more likely to accept it. This assumption was supported by Madanat et al. [38] who showed that travelers with positive attitude towards route diversion are more likely to comply with traffic information. Therefore, we propose:

H4: Attitude towards route diversion has a positive effect on the perceived usefulness of VMS.
H5: Attitude towards route diversion has a positive effect on the intention to use VMS.

### 2.5 Information quality

The concept of information quality used in this study was introduced by DeLone and McLeane [39] who theorized that information quality is a major determinant of the success of an information system. The concept was then adopted and validated by several studies on user acceptance of new information systems [22,40,41]. Information quality is usually characterized by accuracy, completeness, timeliness and display format [42]. Previous research on driver response to traffic information showed that these characteristics are the main determinants of reliance on and compliance with traffic information. Ben-Elia et al. [43] showed that providing travelers with accurate information could increase the compliance rate. Providing accurate information helps travelers make good decisions and improve their travel planning, which enhances their perception of usefulness. Completeness is also a requirement for the success of traffic information systems as proven by Peeta and Ramos [16] who showed providing travelers with the location of the incident, expected delay and best detour strategy is more effective than providing information on location only. Complete VMS information could then give the travelers all the necessary elements needed to make a decision, which enhances their perception of ease of use. Therefore, we can assume a positive effect of information quality on perceived ease of use and perceived usefulness. Previous studies also supported these relationships [22,27,40]. Thus, we propose:

H6: Information quality has a positive effect on the perceived usefulness of VMS.
H7: Information quality has a positive effect on the perceived ease of use of VMS.

Furthermore, information quality is one of the main components of the system usage-continuous usage dynamic process [39]. Continuous usage results from the comparison between the individual’s expectations of the quality of the system and the actual system performance or outcome. If the outcome meets the individual’s expectations, he/she is likely to continue using it. This argument is supported by the Expectance Confirmation Model [44], which shows how the comparison between pre-purchase expectations and post-purchase performance affects a consumer’s satisfaction, which in turn, affects post-purchase attitudes and continuous usage intention. Drawing from this theory, we can assume that when a traveler has a positive perception of information quality (accurate, complete and timely), he/she is likely to divert in response to VMS. At the end of his/her trip, he/she will compare his/her expectations with the actual outcome of his/her behavior. If the outcome meets his/her expectations (e.g. avoiding congestion, reducing travel time, etc.), he/she is likely to develop positive attitudes towards diverting in response to VMS. Thus, we can assume a positive relationship between information quality and attitude towards route diversion and hypothesize:

H8: Information quality has a positive effect on attitude towards route diversion.

### 2.6 Familiarity with the network

In this study, familiarity with the network is defined by attributes such as “*ability to describe the route to my own house*” or “*ability to drive through local streets*”.

In case of congestion, VMS can redirect travelers to an alternate route to help them save time. However, in order to perform a behavior, the individual also takes into account his/her ability to perform the given behavior [45]. In the context of route diversion, some travelers may be unfamiliar with the alternate route. For such travelers, diverting could lead to getting lost and wasting more time than if they stayed on their initial route. As shown by Kronborg [46], travelers are unwilling to divert if the alternate route may take more time. Furthermore, Ma et al. [6] showed that travelers who are unfamiliar with the alternate route are undecided or unwilling to divert. Thus, travelers who are not familiar with the network would develop negative feelings towards diverting. This is not the case with those who are familiar with the network as they have the ability to switch and easily navigate through any part of the network. Therefore, we assume that travelers who are familiar with the network are more likely to develop a positive attitude towards diverting. We hypothesize the following:

H9: Familiarity with the network has a positive effect on attitude towards route diversion.

Furthermore, ATIS can help travelers reduce uncertainty by providing them with road guidance. However, travelers who are familiar with the network are less likely to experience uncertainty as they know the available alternatives and how to navigate through them. For this reason, they would be able to make their routing decisions on their own without relying on traffic information. Earlier studies have suggested that travelers who are familiar with the network are less likely to comply with road guidance [9,47,48]. In their empirical study on travelers’ compliance with VMS, Zhong et al. [10] found that familiarity with the network reduces the propensity to comply with VMS information. Thus, we can assume that travelers who are familiar with the network would be less likely to find VMS information useful. Therefore, we propose:

H10: Familiarity with the network has a negative effect on the perceived usefulness of VMS.

**Fig 2:**
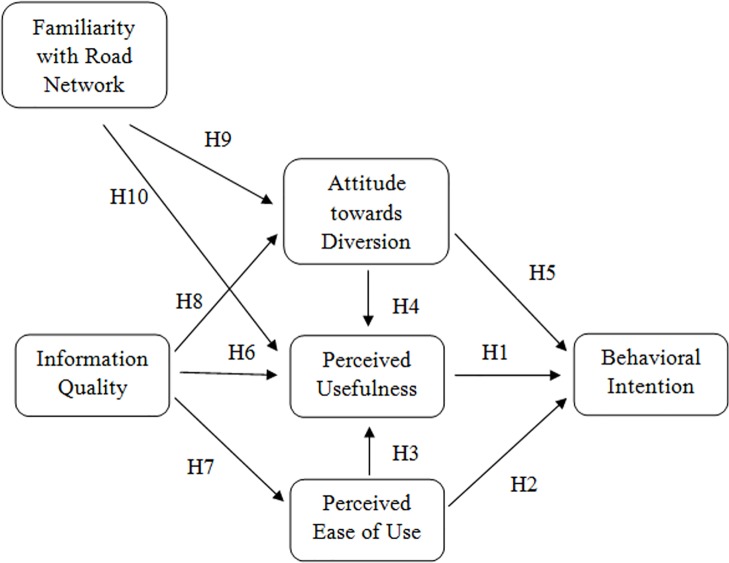
Study framework.

Fig 2 shows the framework developed in this paper.

## Research data and method

### 3.1 Study area

This study took place in Dalian City in the road section between Dalian University of Technology and the city center (Zhongshan Square). This road section consists of two main routes: a local street and an expressway. The whole urban network is equipped with VMS. When the expressway is congested (e.g. during peak hours), the VMS can redirect the drivers to the local street.

### 3.2 Participants

As evidenced by previous research, TAM predicts better future use when the respondent has already experienced the target technology [49,50]. Thus, assuming that people with driver license are more likely to have experienced VMS, this study only interviewed respondents who held a valid driver license. We also asked the respondents to state their response based on their experience with VMS. 800 questionnaires were distributed during an on-site survey and, after elimination of incomplete responses, 762 samples were obtained, indicating a response rate of 95.25%. All participants gave their written consent to participate in the survey and provided some personal information such as their mobile phone number. The written consents along with the study were approved by the Ethics Committee of Dalian University of Technology. Table 1 summarizes the socioeconomic characteristics of the respondents. About 64.57% of the 762 respondents were male, and 35.43% were female. The majority of the respondents were aged between 18 and 30 (58.66%), followed by those aged between 31 and 50 years old (36.22%). Regarding their income, most of the respondents had low income (59.97%), or mid-level income (31.36%). Of the 762 respondents, 47,51% had driving years between 1 and 5 years, followed by those with more than 5 years of driving experience (29.13%). 16.93% of the respondents use *traffic information only* for road guidance, 16.01% use their *experience only*, while 62.86% combined traffic information with their own experience.

**Table 1:** Demographic profile of the respondents.

Attributes	Sub-groups	Frequency(N = 762)	Percentage (%)
Gender	Male	492	64.57
	Female	270	35.43
Age group	18–30	447	58.66
	31–50	276	36.22
	Over 50	39	5.12
Education level	High School and lower	43	5.64
	Associate	137	17.98
	Bachelor	409	53.67
	Graduate	173	22.7
Occupation	Government Worker	140	18.37
	Private Company	326	42.78
	Student	203	26.64
	Other	93	12.2
Marital status	Single	373	48.95
	Married Without Children	81	10.63
	Married With Children	308	40.42
Monthly income	Less than 5,000 RMB	457	59.97
	5,000–10,000 RMB	239	31.36
	More than 10,000 RMB	66	8.66
Driving years	Less than 1 year	178	23.36
	1–5 years	362	47.51
	More than 5 years	222	29.13
Driving Style	Static	32	4.2
	Information-Based	129	16.93
	Experience-Based	122	16.01
	Information-Experience-Based	479	62.86

### 3.3 Measurement scales

The measurement items were selected after a careful review of the literature on behavioral science, user acceptance of new information technology and drivers’ response to traffic information. All items were validated in previous studies. In order the verify the wording and relevance of the selected items in the present study, we interviewed three ITS experts from the local transportation department and two college professors from the School of Transportation and Logistics at Dalian University of Technology. With the help of the aforementioned experts, 20 items were selected for the pilot survey. The questionnaire was then translated into Chinese language by two English professors and an ITS expert. The pilot survey was conducted in April 2017 and helped to further evaluate the validity and wording of the chosen items. 92 respondents (42 staff members and 50 graduate students) were interviewed and the results were used to refine the wording and delete the least relevant items. At the end of this process, nineteen items remained for the final survey.

All items were measured on a 5-point Likert scale ranging from “extremely disagree” to “extremely agree” and “neutral” as mid-point anchor. As recommended by Davis and Venkatesh [33], items measuring the same construct were grouped together. Davis and Venkatesh [33] showed that grouping items is more convenient for the respondents and has no carryover effects. A detailed list of the items is provided in Table 2.

**Table 2:** Attitudinal indicators.

Construct	Items	Wording	Source
Perceived Usefulness	PU1	Using VMS information helps me in avoiding congestion	[18,27,29]
	PU2	Using VMS information helps me in arriving to my destination on time	
	PU3	Using VMS information helps me make better routing and departure time choices	
	PU4	Overall, I find VMS information useful	
Perceived Ease of Use	PEOU1	Using VMS information does not require a lot of mental effort	[18]
	PEOU2	It is easy to learn how to use VMS information	
	PEOU3	VMS information is easy to understand	
	PEOU4	Overall, I find VMS information easy to use	
Information Quality	IQ1	VMS provides accurate traveler information	[27,42]
	IQ2	VMS provides complete traveler information	
	IQ3	VMS provides timely traveler information	
Behavioral Intention	BI1	I would consider using VMS information as long as it is available	[17,21]
	BI2	I will very likely use VMS information if it is available	
	BI3	I would recommend others to use VMS information for their trips	
Attitude towards Route Diversion	ATT1	For my commutes, I often change my planned route	[38]
	ATT2	I am willing to divert in order to avoid traffic congestion	
Familiarity with Road Network	FAM1	I can describe familiar routes	[51]
	FAM2	I can describe the route to my own house	
	FAM3	I am familiar with driving through local streets	

### 3.4 Data processing method

The statistical software R (lavaan package) was used to test the hypotheses developed in Section 2. The model was built using the two-stage approach recommended by Anderson and Gerbing [52]. The first stage consists of building a confirmatory factor analysis (CFA) to assess the validity and reliability of the items. Once the CFA is deemed adequate, the causal relationships are simultaneously tested using Structural Equation Modeling (SEM).

## Results

### 4.1 Normality check

In order to verify which estimation method would be more suitable for our data, we performed a normality check by computing the univariate kurtosis and skewness. As a rule of thumb, absolute values of skew indexes larger than 3 and absolute values of kurtosis indexes larger than 10 are indication that there is severe violation of normality [53]. In this study, the skew indexes range from -1.161 to -0.168, the kurtosis indexes range from 1.929 to 4.753. Thus, we can assume there is no extreme violation of normality. Assuming that the data approximately follow a normal distribution, we adopt Maximum Likelihood estimation method to build the CFA and SEM used in this Section. A detailed list of the univariate kurtosis and skewness for each indicator is provided in S3 File.

### 4.2 Measurement model

#### 4.2.1 Exploratory factor analysis

We first performed an Exploratory Factor Analysis (EFA) to check the factor structure of the empirical data. The EFA was built using Principal Axis Factoring (PAF) method and promax rotation. Using a cut-off value of 0.3, we found no sign of cross-loading and the factor structure matches the model used in this study. Thus, we can use these indicators to perform our CFA. The results of the EFA (standardized factor loadings) are provided in S4 File.

#### 4.2.2 Confirmatory factor analysis

In order to evaluate the reliability and validity of the constructs, we computed the composite reliability and Average Variance Extracted (AVE). For item reliability, the composite reliability must be larger than 0.6 [54]. As shown in Table 3, all composite reliability measures ranged from 0.804 to 0.893. Thus, our model meets the recommendation for item reliability. Convergent validity is demonstrated when different items are used to measure the same construct, and the scores from the different items are strongly correlated. To ensure convergent validity, all factor loadings should be significant [52] and larger than 0.5 [55]. Table 3 shows that the factor loadings of all items are highly significant (p<0.001) and larger than 0.5. Discriminant validity evaluates if the constructs are adequately distinguishable from one another. Discriminant validity is demonstrated if the square root of the AVE for a construct is larger than its correlations with other constructs [56]. Table 4 shows that the measurement model meets the requirement for discriminant validity.

**Table 3:** Validity and reliability of the measurement model.

Constructs	Indicators	Mean	SD	AVE	λ	P Value	CR
Perceived Usefulness	PU1	4.538	0.629	0.675	0.770	<0.001	0.892
	PU2	4.471	0.684		0.834	<0.001	
	PU3	4.491	0.684		0.803	<0.001	
	PU4	4.541	0.597		0.876	<0.001	
Perceived Ease of Use	PEOU1	4.109	0.835	0.678	0.759	<0.001	0.893
	PEOU2	3.999	0.826		0.849	<0.001	
	PEOU3	4.144	0.727		0.863	<0.001	
	PEOU4	4.161	0.730		0.818	<0.001	
Information Quality	IQ1	3.938	0.940	0.678	0.852	<0.001	0.863
	IQ2	3.655	1.082		0.854	<0.001	
	IQ3	4.073	0.874		0.762	<0.001	
Behavioral Intention	BI1	4.429	0.603	0.695	0.899	<0.001	0.804
	BI2	4.412	0.626		0.889	<0.001	
	BI3	4.210	0.703		0.698	<0.001	
Attitude towards Route Diversion	ATT1	4.315	0.716	0.527	0.738	<0.001	0.810
	ATT2	4.358	0.662		0.714	<0.001	
Familiarity with Road Network	FAM1	4.164	0.801	0.681	0.825	<0.001	0.871
	FAM2	4.181	0.795		0.873	<0.001	
	FAM3	4.184	0.849		0.774	<0.001	

SD, Standard Deviation; AVE, Average Variance Extracted; λ, Factor Loadings; CR, Composite Reliability.

**Table 4:** Inter-construct correlations as discriminant validity (square root of AVE in diagonals).

	**PU**	**PEOU**	**IQ**	**BI**	**ATT**	**FAM**
PU	**0.821**					
PEOU	0.488	**0.823**				
IQ	0.441	0.563	**0.823**			
BI	0.626	0.599	0.452	**0.834**		
ATT	0.466	0.614	0.567	0.660	**0.726**	
FAM	0.262	0.589	0.412	0.475	0.555	**0.825**

PU, Perceived Usefulness; PEOU, Perceived Ease of Use; IQ, Information Quality; BI, Behavioral Intention; ATT, Attitude towards Route Diversion; FAM, Familiarity with the Road Network.

Square roots of AVEs are in diagonals.

The fit indexes evaluate how well the measurement model represents the sample. The p-value of the Chi-square was 0.000 (χ^2^ = 462.777, df = 126). The p-value should be larger than 0.05 for good model fit. However, the Chi-square is too sensitive to large sample size; therefore it generally rejects well-fitting models when large sample sizes are used [57]. One way to minimize the effect of sample size is by using the normed Chi-square (χ^2^/df) [58]. Wheaton et al. [58] recommended a normed Chi-square between 1 and 5 for an acceptable fit. The value obtained in this study was 3.67, indicating an acceptable fit. The Root Mean Square Error of Approximation (RMSEA) and Standardized Root Mean Square Residual (SRMR) were 0.059 and 0.041, respectively. An RMSEA less than 0.07 and SRMR less than 0.08 are considered an indication of acceptable fit [59,60]. Thus, the SRMR and RMSEA were within the recommended range. The Comparative Fit Index (CFI), Normed-Fit Index (NFI) and Tucker-Lewis Index (TLI) were 0.964, 0.951 and 0.951, respectively, exceeding the cut-off value of 0.95 recommended by Hu and Bentler [60].

In summary, all relevant fit indexes and reliability measures were within the recommended ranges; indicating that the measurement model satisfied all criteria for model fit, construct reliability and validity. Therefore, the model could be used to test the causal relationships hypothesized in Section 2.

### 4.3 Path analysis

**Fig 3:**
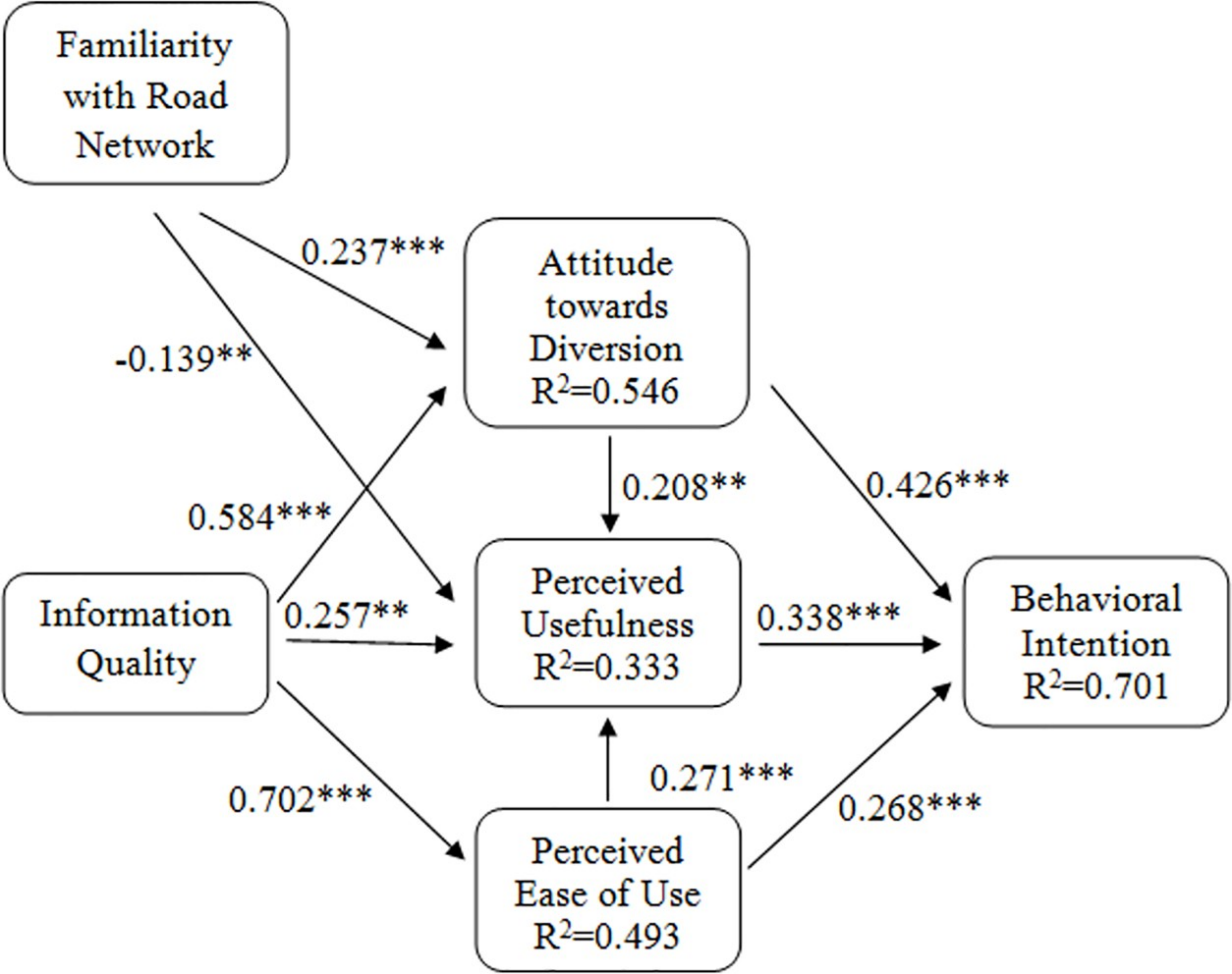
Results of path analysis. **p<0.01; ***p<0.001.

Similar to the measurement model, all fit indexes for the structural model were within the recommended ranges (χ^2^/df = 3.82, CFI = 0.963, NFI = 0.951, TLI = 0.95, RMSEA = 0.061, SRMR = 0.054), providing support for model fit. Fig 3 shows the results of the path analysis with standardized coefficients. Regarding the traditional TAM, perceived ease of use has a significant and positive impact on perceived usefulness (*β* = 0.271, *p* < 0.001) and behavioral intention (*β* = 0.268, *p* < 0.001), providing support for H2 and H3. Perceived usefulness positively determines behavioral intention (*β* = 0.338, *p* < 0.001). We could, therefore, accept H1. These results show that all hypotheses regarding the basic TAM are supported. Thus, TAM could be used as a basis for our extended model. Attitude towards route diversion positively affects perceived usefulness (*β* = 0.208, *p* < 0.01) and behavioral intention (*β* = 0.426, *p* < 0.001). These findings support H4 and H5. Information quality has a positive effect on perceived usefulness (*β* = 0.257, *p* < 0.01), perceived ease of use (*β* = 0.702, *p* < 0.001) and attitude towards route diversion (*β* = 0.584, *p* < 0.001), providing empirical support for H6, H7 and H8, respectively. Familiarity with the road network has a positive effect on attitude towards route diversion (*β* = 0.237, *p* < 0.001) and a negative impact on perceived usefulness (*β* = ˗0.139, *p* < 0.01), supporting H9 and H10, respectively. Familiarity with the road network and information quality represent 54.6% of the variance in attitude towards route diversion. Perceived ease of use, information quality, attitude towards route diversion and familiarity with road network represent 33.3% of the variance in perceived usefulness. Information quality explains 49.3% of the variance in perceived ease of use. Perceived ease of use, perceived usefulness and attitude towards route diversion collectively explain 70.1% of the variance in behavioral intention. Next section discusses the results of this study in detail by providing empirical support from previous studies and identifying the academic and practical implications of our findings.

## Discussion

### 5.1 Implications

For the traditional TAM constructs, perceived usefulness has a stronger effect on behavioral intention. Compared to other TAM studies on user acceptance of road guidance systems, this finding is contrary to Chen and Chen [29] and Xu et al. [28] who found that perceived usefulness had little to no effect on behavioral intention, but is supported by Ghazizadeh et al. [25] and Larue et al. [26]. These inconsistencies prove that the effect of perceived usefulness and perceived ease of use depends on the sample, study context, and technology under consideration. For example, Xu et al. [28] interviewed road users from Nanjing, China, while this study targeted drivers in Dalian, China and found different effects. Thus, even in the same country, the behavior may be different between two different cities. Context is also a major determinant as many studies have shown that perceived ease of use is stronger in mandatory settings [61]. However, the use of traffic information is not mandatory (travelers have the option to comply or ignore the guidance); therefore it is not surprising to find perceived usefulness as the stronger determinant of behavioral intention. In terms of practical implication, the strong effect of perceived usefulness on behavioral intention indicates that ITS designers should take into account the context of use of the given technology before implementation. If use is voluntary, as in the case of VMS, then they should put more emphasis on individuals’ perception of usefulness. This could be done by (a) improving user perception of the determinants of perceived usefulness such as information quality and/or (b) local authorities and ITS developers could organize campaigns that aim to promote the benefits of VMS.

Information quality has a higher effect on perceived ease of use than perceived usefulness, which is inconsistent with many previous TAM studies [22,27,40]. However, this is similar to findings by Kuo and Lee [41] and could be explained by the specificity of travelers’ behavior in response to VMS. VMS are installed along the road and drivers have the complex task of reading, processing the information and making a decision in little amount of time, all while driving. Thus, having a VMS information of poor quality could increase the complexity of the driving task and lead to distraction, which, in turn, could threaten their safety [62]. For this reason, information quality plays a very important role in travelers’ perception of ease of use. As for the managerial implications, ATIS providers should make more efforts and use the new technological advancements to provide accurate, complete and timely information. For example, complete information on the occurrence of traffic accidents should also contain the type of incident (e.g. accident, congestion, etc.), the exact location of the incident, the expected delay, and best routing strategy. As far as timeliness is concerned, the information should be provided at the appropriate time, when it is most needed. For example, when the information is displayed too close to the incident site, the driver may not have enough time to react. Alternatively, when the information is displayed too far from the incident site, the driver may find it irrelevant. Providing such information would for sure increase the reliability of VMS, which could result in a higher acceptance.

The inclusion of attitude towards route diversion is new in TAM studies; therefore there is no empirical evidence from the TAM literature to back this finding. However, research on driver’s response to traffic information has demonstrated that attitude towards diversion has a positive effect on driver’s compliance with traffic information [38]. Furthermore, attitude is the strongest predictor of intention and second strongest determinant of perceived usefulness. This indicates that attitude towards diversion is one of the main determinants of travelers’ acceptance of VMS. Thus, for future studies examining the effects of road guidance systems, we recommend the inclusion of attitude towards route diversion as determinant of behavioral intention and perceived usefulness.

Familiarity with the network has a positive effect on attitude towards road diversion and a negative effect on perceived usefulness. This supports our assumption that travelers who are familiar with the network can assess the current traffic conditions and make the decision to divert or not without relying on traffic information. Given their positive attitude towards road diversion, they would divert to the alternate route if necessary. However, travelers who are not familiar with the network are more likely to need road guidance [10], but would hesitate to drive through unfamiliar routes. In order to convince these drivers to divert in response to VMS, the message could be displayed with a map that describes the layout of all available alternatives. In this case, the traffic information would serve as a road guidance and navigation system. The types of VMS that can display traffic information as a map already exist and are known as GRIP (Graphical Route Information Panel). GRIPs have already proven to be more effective than the traditional VMS [1,2]. Adopting such new technology could help improve travelers’ acceptance of VMS.

### 5.2 Limitations

This study suffers from some limitations, which could be addressed in future studies. First, the model was developed using cross-sectional data. However, previous studies have shown that attitudes and perception may change over time [18]. Actual system use also may change people’s perceptions and affect long-term adoption [39]. Thus, a longitudinal study using different points in time could help understand the evolution of perceptions and attitudes towards the long-term use of VMS. For example, by using latent growth curve analysis, we can investigate how travelers’ attitudes and perceptions have evolved after the implementation of new policies and whether these changes are due to the new policies or other factors. Second, though *attitude towards route diversion* had a strong positive effect on perceived usefulness and behavioral intention, its use in TAM is new. More research is needed to investigate the effects of this construct before our findings could be generalized. Third, despite a pretty high explanatory power, the model did not use any TPB (Theory of Planned Behavior) construct. However, research in the driving assistance domain that combined TAM and TPB found a higher explanatory power. For example, Chen and Chen [30], found that a combined TAM-TPB explained 93% of the intention to use telematics. Thus, in addition to the constructs used in this study, incorporating the TPB constructs could help improve our model. Finally, TAM is too general and does not apply to specific traffic conditions. Previous research has shown that current traffic conditions such as delay, travel time on alternate routes and number of signalized intersections may also affect travelers’ response to ATIS [4,16,43,63]. Thus, an Integrated Choice and Latent Variable (ICLV) model combining TAM (as latent variable sub-model) and current traffic conditions (as attributes in the choice sub-model) could help better understand travelers’ decision-making process. Our future research efforts will take these limitations into consideration.

## Supporting information

S1 File Data used in this study. Indicators and Socioeconomic Characteristics of Respondents.
(XLSX)

S2 File Survey used in this study. Survey questionnaire in English and Chinese Language.
(PDF)

S3 File Skewness and kurtosis indexes. Skewness and kurtosis indexes for normality check.
(DOCX)

S4 File Results of exploratory factor analysis. (DOCX)
