# Supplementary material for: An extension of the technology acceptance model for understanding travelers’ adoption of variable message signs
Source: PLoS One. 2019 Apr 25;14(4):e0216007. doi: 10.1371/journal.pone.0216007 (PMC6483246; doi:10.1371/journal.pone.0216007)
Supplement: S2 File — Survey questionnaire in English and Chinese Language. (PDF) [file pone.0216007.s002.pdf]

可变信息板（ＶＭＳ）反应与路径选择行为调查

尊敬的先生/女士，您好！

我们正在进行一项关于交通信息与行为的调查，想邀请您用几分钟时间帮忙填答这份问卷。本次调查的目的是研究道路可变信息板（ＶＭＳ） 对驾驶员的影响，您的回答将有助于改善可变信息标志的设计，进而有助于缓解交通拥堵。本次调查完全匿名，调查结果仅用于学术研究，不会泄露您的任何个人信息，请放心作答。

您认真如实的填写，对取得正确的研究成果非常重要。感谢您的支持与帮助！

第一部分 对可变信息板（ＶＭＳ）的反应与路径选择

（请在选项的“□”里划 “√” 作答，每题仅限选一项）

1.	VMS 有助于避免交通拥堵	□非常反对 □反对 □中立 □同意 □非常同意
2.	VMS 有助于按时到达目的地	□非常反对 □反对 □中立 □同意 □非常同意
3.	VMS 有助于选择更好的路线和出发时间	□非常反对 □反对 □中立 □同意 □非常同意
4.	总体而言，VMS 是有用的	□非常反对 □反对 □中立 □同意 □非常同意
5.	使用 VMS 不需要费太多精力	□非常反对 □反对 □中立 □同意 □非常同意
6.	学习使用 VMS 是容易的	□非常反对 □反对 □中立 □同意 □非常同意
7.	VMS 提供的信息是容易理解	□非常反对 □反对 □中立 □同意 □非常同意
8.	总体而言，VMS 是容易使用的	□非常反对 □反对 □中立 □同意 □非常同意
9.	VMS 提供准确信息	□非常反对 □反对 □中立 □同意 □非常同意
10.	VMS 提供与交通相关的完整信息	□非常反对 □反对 □中立 □同意 □非常同意
11.	VMS 提供及时的出行者信息	□非常反对 □反对 □中立 □同意 □非常同意
12.	只要条件允许，我会考虑使用 VMS	□非常反对 □反对 □中立 □同意 □非常同意
13.	只要条件允许，我很可能会使用 VMS	□非常反对 □反对 □中立 □同意 □非常同意
14.	我会推荐他人出行时使用 VMS	□非常反对 □反对 □中立 □同意 □非常同意

15. **我经常在出行途中改变原计划路线** ☐非常反对 ☐反对 ☐中立 ☐同意 ☐非常同意

16. **为避免交通拥堵，我愿意改变出行路线** ☐非常反对 ☐反对 ☐中立 ☐同意 ☐非常同意

17. **我可以描述熟悉路线上的交通状况** ☐非常反对 ☐反对 ☐中立 ☐同意 ☐非常同意

18. **我可以描述出回家路上的交通状况** ☐非常反对 ☐反对 ☐中立 ☐同意 ☐非常同意

19. **我对开车穿过街道的小路很熟悉** ☐非常反对 ☐反对 ☐中立 ☐同意 ☐非常同意

第二部分 驾驶员特性

1. **性别：**

☐男 ☐女

2. **年龄：**

☐18-30 岁 ☐31-50 岁 ☐50 岁以上

3. **您的学历：**

☐高中及以下 ☐专科 ☐本科 ☐研究生

4. **婚姻状况：**

☐未婚 ☐已婚无子女 ☐已婚有子女

5. **收入情况（月收入）：**

☐5000 元以下 ☐5000-10000 元 ☐10000 元以上

6. **职业：**

☐公务员、事业工作人员 ☐企业工作人员 ☐学生 ☐其他

7. **是否有驾照：**

☐是 ☐否

8. **驾龄：**

☐1 年以下 ☐1-5 年 ☐5 年以上

9. **路径选择方式（仅限选一项）：**

☐信息型选择 ☐经验型选择 ☐经验和信息型选择 ☐固定选择

本问卷到此结束，非常感谢您的合作！

如有疑问，请联系[redacted]

联系电话：[redacted]

VARIABLE MESSAGE SIGNS AND ROUTE SWITCHING BEHAVIOR

Dear Madam/Sir,
We are conducting a survey on traffic information and travel behavior. We would like to invite you take a few minutes to fill out this questionnaire. The purpose of this survey is to examine the impact of Variable Message Signs (VMS) on driver behavior. Your answers will help improve the design of variable message signs and thus alleviate traffic congestion. Your responses will be anonymous and will never be linked to you personally. The results of this survey are only used for academic research.

Your responses are of the utmost importance to us for obtaining correct findings. Thank you for your cooperation!

Part 1: Attitudes and Perceptions towards VMS and route diversion

Please give your degree of agreement with the following statements
（For each statement, please check one box）

1.	**Using VMS information helps me in avoiding congestion**	Strongly Disagree ☐	Disagree ☐	Neutral ☐	Agree ☐	Strongly Agree ☐
2.	**Using VMS information helps me in arriving to my destination on time**	Strongly Disagree ☐	Disagree ☐	Neutral ☐	Agree ☐	Strongly Agree ☐
3.	**Using VMS information helps me make better routing and departure time choices**	Strongly Disagree ☐	Disagree ☐	Neutral ☐	Agree ☐	Strongly Agree ☐
4.	**Overall, I find VMS information useful**	Strongly Disagree ☐	Disagree ☐	Neutral ☐	Agree ☐	Strongly Agree ☐
5.	**Using VMS information does not require a lot of mental effort**	Strongly Disagree ☐	Disagree ☐	Neutral ☐	Agree ☐	Strongly Agree ☐
6.	**It is easy to learn how to use VMS information**	Strongly Disagree ☐	Disagree ☐	Neutral ☐	Agree ☐	Strongly Agree ☐
7.	**VMS information is easy to understand**	Strongly Disagree ☐	Disagree ☐	Neutral ☐	Agree ☐	Strongly Agree ☐
8.	**Overall, I find VMS information easy to use**	Strongly Disagree ☐	Disagree ☐	Neutral ☐	Agree ☐	Strongly Agree ☐
9.	**VMS provides accurate traveler information**	Strongly Disagree ☐	Disagree ☐	Neutral ☐	Agree ☐	Strongly Agree ☐

10.	**VMS provides complete traveler information**	Strongly Disagree ☐	Disagree ☐	Neutral ☐	Agree ☐	Strongly Agree ☐
11.	**VMS provides timely traveler information**	Strongly Disagree ☐	Disagree ☐	Neutral ☐	Agree ☐	Strongly Agree ☐
12.	**I would consider using VMS information as long as it is available**	Strongly Disagree ☐	Disagree ☐	Neutral ☐	Agree ☐	Strongly Agree ☐
13.	**I will very likely use VMS information if it is available**	Strongly Disagree ☐	Disagree ☐	Neutral ☐	Agree ☐	Strongly Agree ☐
14.	**I would recommend others to use VMS information for their trips**	Strongly Disagree ☐	Disagree ☐	Neutral ☐	Agree ☐	Strongly Agree ☐
15.	**For my commutes, I often change my planned route**	Strongly Disagree ☐	Disagree ☐	Neutral ☐	Agree ☐	Strongly Agree ☐
16.	**I am willing to divert in order to avoid traffic congestion**	Strongly Disagree ☐	Disagree ☐	Neutral ☐	Agree ☐	Strongly Agree ☐
17.	**I can describe familiar routes**	Strongly Disagree ☐	Disagree ☐	Neutral ☐	Agree ☐	Strongly Agree ☐
18.	**I can describe the route to my own house**	Strongly Disagree ☐	Disagree ☐	Neutral ☐	Agree ☐	Strongly Agree ☐
19.	**I am familiar with driving through local streets**	Strongly Disagree ☐	Disagree ☐	Neutral ☐	Agree ☐	Strongly Agree ☐

Part 2: Individual Characteristics

1. **Gender：**

   ☐ Male ☐Female

2. **Age：**

   ☐18-30 ☐31-50 ☐Over 50

3. **Education Level：**

   ☐High School and Under ☐Associate ☐Bachelor ☐Graduate

4. **Marital Status：**

   ☐Single ☐Married without Children ☐Married with Children

5. **Monthly Income：**

☐Less than 5000 RMB ☐5000-10000 RMB ☐More than 10000 RMB

6. **Occupation：**

☐Government ☐Private Company ☐Student ☐Other

7. **Do you have a driver license?**

☐Yes ☐No

8. **Driving years：**

☐Less than 1 year ☐1-5 years ☐More than 5 years

9. **Route Choice Style:**

☐Static ☐Information-based ☐Experience-based ☐Information-experience-based

Thank you for your cooperation！

If you have any question，please contact [redacted]

Phone number：[redacted]
