# Supplementary material for: An extension of the technology acceptance model for understanding travelers’ adoption of variable message signs
Source: PLoS One. 2019 Apr 25;14(4):e0216007. doi: 10.1371/journal.pone.0216007 (PMC6483246; doi:10.1371/journal.pone.0216007)
Supplement: S3 File — Skewness and kurtosis indexes for normality check. (DOCX) [file pone.0216007.s003.docx]

| **Constructs** | **Indicators** | **Kurtosis** | **Skewness** |
| --- | --- | --- | --- |
| Perceived Usefulness | PU1 | 3.811 | -1.156 |
|  | PU2 | 3.599 | -1.092 |
|  | PU3 | 3.719 | -1.161 |
|  | PU4 | 2.839 | -0.917 |
| Perceived Ease of Use | PEOU1 | 3.209 | -0.736 |
|  | PEOU2 | 2.484 | -0.390 |
|  | PEOU3 | 2.495 | -0.392 |
|  | PEOU4 | 2.642 | -0.462 |
| Information Quality | IQ1 | 2.326 | -0.466 |
|  | IQ2 | 1.929 | -0.168 |
|  | IQ3 | 2.679 | -0.638 |
| Behavioral Intention | BI1 | 3.044 | -0.644 |
|  | BI2 | 3.146 | -0.709 |
|  | BI3 | 2.775 | -0.500 |
| Attitude towards Route Diversion | ATT1 | 3.168 | -0.783 |
|  | ATT2 | 4.272 | -0.927 |
| Familiarity with Road Network | FAM1 | 4.753 | -1.040 |
|  | FAM2 | 4.608 | -1.057 |
|  | FAM3 | 4.362 | -1.120 |
