# Supplementary material for: An extension of the technology acceptance model for understanding travelers’ adoption of variable message signs
Source: PLoS One. 2019 Apr 25;14(4):e0216007. doi: 10.1371/journal.pone.0216007 (PMC6483246; doi:10.1371/journal.pone.0216007)
Supplement: S4 File — (DOCX) [file pone.0216007.s004.docx]

**Exploratory Factor Analysis using Principal Axis Factoring; rotation Promax; Standardized factor loadings.**

|  | **Factor1** | **Factor2** | **Factor3** | **Factor4** | **Factor5** | **Factor6** |
| --- | --- | --- | --- | --- | --- | --- |
| PU1 | 0.86 |  |  |  |  |  |
| PU2 | 0.91 |  |  |  |  |  |
| PU3 | 0.76 |  |  |  |  |  |
| PU4 | 0.8 |  |  |  |  |  |
| PEOU1 |  | 0.75 |  |  |  |  |
| PEOU2 |  | 0.89 |  |  |  |  |
| PEOU3 |  | 0.78 |  |  |  |  |
| PEOU4 |  | 0.85 |  |  |  |  |
| IQ1 |  |  | 0.83 |  |  |  |
| IQ2 |  |  | 0.92 |  |  |  |
| IQ3 |  |  | 0.77 |  |  |  |
| ATT1 |  |  |  |  |  | 0.64 |
| ATT2 |  |  |  |  |  | 0.77 |
| FAM1 |  |  |  | 0.79 |  |  |
| FAM2 |  |  |  | 0.88 |  |  |
| FAM3 |  |  |  | 0.75 |  |  |
| BI1 |  |  |  |  | 0.97 |  |
| BI2 |  |  |  |  | 0.86 |  |
| BI3 |  |  |  |  | 0.42 |  |
